# CircRNA_09505 aggravates inflammation and joint damage in collagen-induced arthritis mice via miR-6089/AKT1/NF-κB axis

**DOI:** 10.1038/s41419-020-03038-z

**Published:** 2020-10-07

**Authors:** Jinghan Yang, Min Cheng, Bingjie Gu, Jinghua Wang, Shushan Yan, Donghua Xu

**Affiliations:** 1 Department of Rheumatology & Central Laboratory of the First Affiliated Hospital, Weifang Medical University, Weifang, 261053 China; 2 Department of Physiology, Clinical Medicine College, Weifang Medical University, Weifang, 261053 China; 3 Department of Rheumatology and Immunology, Nanjing First Hospital, Nanjing Medical University, Nanjing, 210006 China; 4 Department of Gastrointestinal and Anal Diseases Surgery of the Affiliated Hospital, Weifang Medical University, Weifang, 261053 China

**Keywords:** Non-coding RNAs, Rheumatoid arthritis

## Abstract

A number of circular RNAs (circRNAs) have been implicated in rheumatoid arthritis (RA) pathogenesis; however, little is known about their function and hidden molecular mechanism in immune and inflammation regulation. We investigated the role and the underlying mechanism of circRNA_09505 in RA in this study. Real-time PCR and fluorescence in situ hybridization (FISH) are adopted to estimate the quantitative expression and localization of circRNA_09505 in macrophages. The altering effect of circRNA_09505 on inflammation is investigated in vitro and in vivo by use of macrophage cell models and collagen-induced arthritis (CIA) mice. Luciferase reporter assay and RNA-binding protein immunoprecipitation (RIP) are used to confirm the circRNA_09505/miR-6089 ceRNA network predicted by bioinformatics analysis. Compared with controls, the expression of circRNA_09505 is upregulated in peripheral blood mononuclear cells (PBMCs) from patients with RA. The proliferation and cell cycle are significantly promoted when circRNA_09505 is upregulated in macrophages, whereas knockdown of circRNA_09505 inhibits macrophage proliferation and cell- cycle progression. Besides, circRNA_09505 can act as a miRNA sponge for miR-6089 in macrophages, and promote the production of TNF-α, IL-6, and IL-12 through ceRNA mechanism. Moreover, AKT1 is a direct target of miR-6089. CircRNA_09505 can promote AKT1 expression by acting as a miR-6089 sponge via IκBα/NF-κB signaling pathway in macrophages. Most interestingly, knockdown of circRNA_09505 significantly alleviates arthritis and inflammation in vivo in CIA mice. These data support the hypothesis that circRNA_09505 can function as a miR-6089 sponge and regulate inflammation via miR-6089/AKT1/NF-κB axis in CIA mice.

## Introduction

Rheumatoid arthritis (RA) is a systemic autoimmune disease with unclear pathogenesis^1^. Inflammation and immunological disorders caused by macrophage, B, and T lymphocytes contribute to RA^2^. Abnormal phenotype and function of those cells play crucial roles in regulating autoimmunity and inflammation. Both innate and adaptive immune reactions are involved in the pathogenesis of RA. Macrophages are essential cells that promote systemic autoimmune disorders and chronic joint inflammation by linking innate and adaptive immunity in RA^3,4^. Increased level of macrophages in inflammatory lesions is a critical feature of RA, which infiltrates in synovial tissues and causes joint erosion. Macrophages have plasticity and pluripotency, which can differentiate into pro-inflammatory macrophages (called M1) and anti-inflammatory macrophages (called M2) under diverse microenvironments^5,6^. Autoimmune disorders and excessive inflammatory responses perpetuate polarization of macrophages to a pro-inflammatory phenotype (M1), and thereby contribute to RA. As a result, depletion of macrophages from inflamed tissue is a promising strategy for RA treatment.

Mounting data have implicated that noncoding RNAs (ncRNAs) primarily including microRNA (miRNA), circular RNA (circRNA), and long ncRNA (lncRNA) play critical roles in mediating inflammation and immune regulation in autoimmune diseases^7–9^. Most interestingly, some ncRNAs encapsulated in extracellular vesicles can be found in peripheral circulation and confer modifying effects on joint lesions and tissue regeneration via cell-to-cell communications in rheumatic diseases^10,11^. CircRNAs are stable ncRNAs with high evolutionary conservation. Emerging evidence has suggested that circRNAs are rich in binding sites of miRNAs and can sponge miRNAs through the competitive endogenous RNA (ceRNA) mechanism in complex biological processes, such as autoimmune imbalance, inflammatory response, and carcinogenesis^12^. Many specifically expressed circRNAs have been identified in RA, some of which regulate RA through ceRNA mechanism^13,14^. Nevertheless, the precise mechanism for circRNA/miRNA ceRNA network in RA has not been fully understood.

We have previously identified the specific profile of circRNA in RA patients by RNA sequencing. CircRNA_09505 is one of the most aberrantly expressed circRNAs in RA, which can also interact with miRNA through ceRNA mechanism predicted by bioinformatics analysis. The aim of this study is to explore the function and molecular mechanism of circRNA_09505 in vitro and in vivo by use of macrophage model and collagen-induced arthritis (CIA) mouse model.

## Results

### Screening differentially expressed circRNAs in RA

**Fig. 1:**
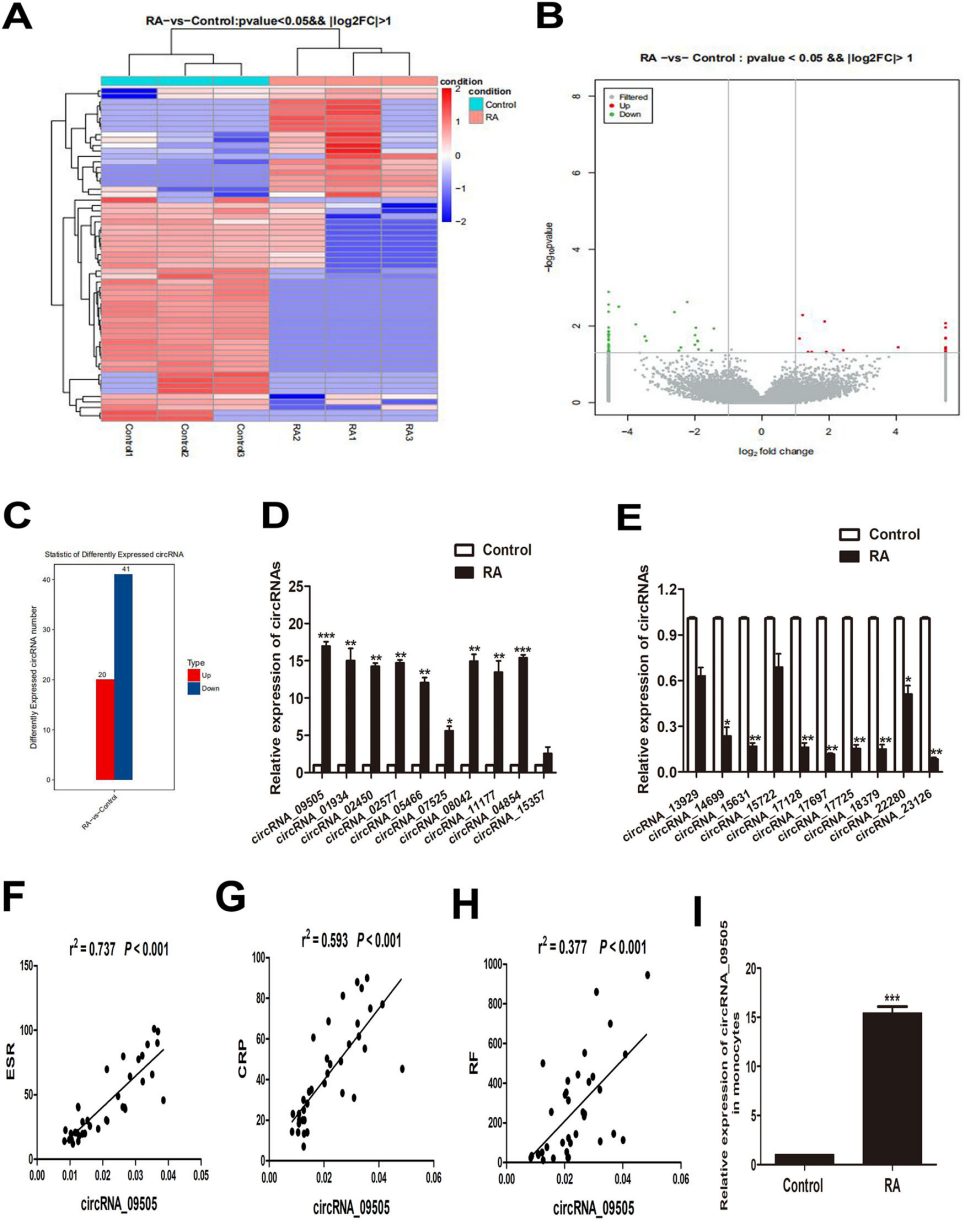
CircRNA_09505 was aberrantly expressed and positively associated with ESR, CRP, and RF in RA. **a** Heat map. **b** Volcano scatter. **c** Column analyses showing differently expressed circRNAs in three RA patients compared with three healthy controls (up/down: 20/41, *P* < 0.05, and |log2FC| > 1 are considered as statistically significant). **d** Real-time PCR confirmed the expression of top 10 upexpressed circRNAs in PBMCs from 36 patients and 30 controls. **e** Real-time PCR confirmed the expression of top 10 downexpressed circRNAs in PBMCs from RA patients (RA/Control: 36/30). **f** CircRNA_09505 was positively associated with ESR in RA (RA: 36). **g** CircRNA_09505 was positively related to CRP in RA (RA: 36). **h** CircRNA_09505 was positively associated with RF in RA (RA: 36). **i** Increased circRNA_09505 expression in monocytes from RA patients compared with healthy controls (RA/Control: 21/18) (*P < 0.05, **P < 0.01, ***P < 0.001; ESR erythrocyte sedimentation rate, CRP C-responsive protein, RF rheumatoid factor).

Differentially expressed circRNAs in peripheral blood mononuclear cells (PBMCs) from three patients and three controls were screened by RNA sequencing. As shown in Fig. 1a–c, there were 20 upregulated and 41 downregulated aberrantly expressed circRNAs in RA. Table 1 presents the top ten most significantly upregulated and downregulated circRNAs differentially expressed in RA, respectively. The top 20 dysregulated circRNAs were screened by RNA sequence and validated in PBMC samples from 36 patients and 30 controls by real-time polymerase chain reaction (PCR). Similar to the results of RNA sequencing, nine circRNAs were significantly upregulated, while eight circRNAs were significantly downregulated in RA PBMCs (Fig. 1d, e). Among them, circRNA_09505 was the most significantly upregulated one in PBMCs from RA patients. Most interestingly, the expression of circRNA_09505 was positively associated with ESR, CRP, and RF levels in serum from RA patients (Fig. 1f–h), suggesting significant relationship between circRNA_09505 expression and RA disease activity. Most importantly, the expression of circRNA_09505 was also found to be significantly increased in peripheral monocytes from RA patients compared with controls (Fig. 1i). GO and KEGG pathway enrichment analysis have suggested the differentially expressed circRNAs, including circRNA_09505 that might exert effects through the classic inflammation-related signaling pathways, such as NF-κB and TNF signaling pathways (Supplementary Fig. 1). Accordingly, we hypothesized that circRNA_09505 played a critical role in mononuclear macrophage-mediated inflammation in RA pathogenesis. In the following experiments, we further investigated the effect of circRNA_09505 and the underlying mechanism in regulating macrophages’ inflammatory response in RA.

**Table 1:** Top ten upregulated and downregulated aberrantly expressed circRNAs in RA PBMCs.

CircRNAs	Fold	Change	*P*
circRNA_09505	18.002	Up	0.011
circRNA_01934	17.442	Up	0.020
circRNA_02450	17.316	Up	0.048
circRNA_02577	17.222	Up	0.036
circRNA_05466	17.215	Up	0.038
circRNA_07525	17.115	Up	0.036
circRNA_08042	16.490	Up	0.040
circRNA_11177	16.455	Up	0.008
circRNA_04854	16.322	Up	0.021
circRNA_15357	16.113	Up	0.040
circRNA_13929	17.991	Down	0.019
circRNA_14699	17.402	Down	0.032
circRNA_15631	17.389	Down	0.046
circRNA_15722	17.168	Down	0.046
circRNA_17128	17.117	Down	0.034
circRNA_17697	16.111	Down	0.017
circRNA_17725	15.088	Down	0.014
circRNA_18379	15.069	Down	0.014
circRNA_22280	14.621	Down	0.040
circRNA_23126	14.002	Down	0.016

### CircRNA_09505 promoted macrophage proliferation and progression of cell cycle

**Fig. 2:**
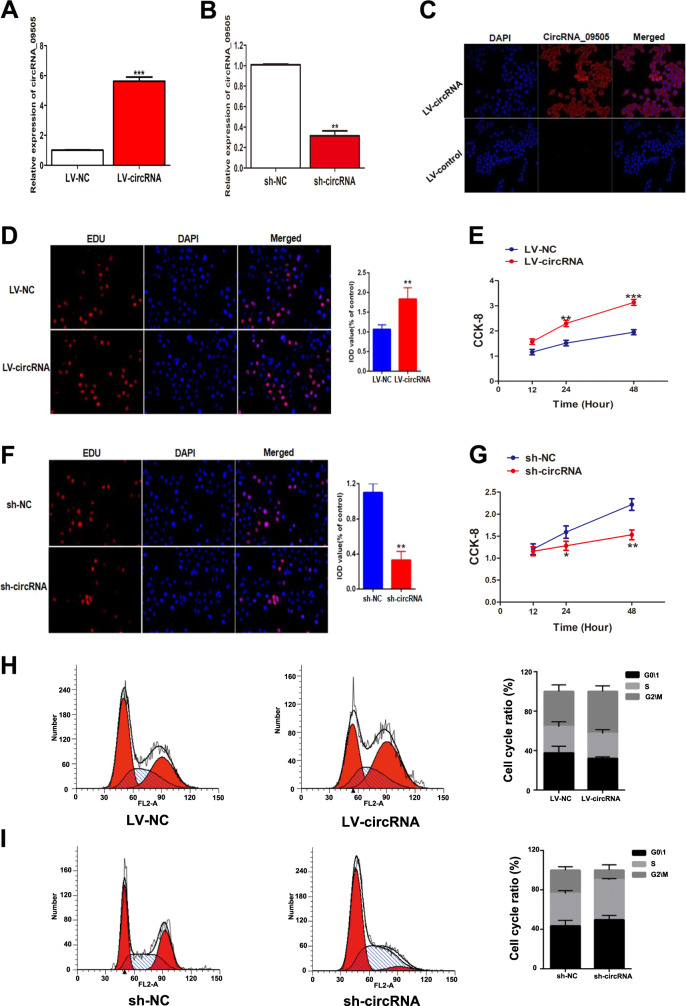
CircRNA_09505 enhanced macrophage proliferation and cell-cycle progression. **a** Real-time PCR showed that circRNA_09505 was increased in LV-circRNA-transfected macrophages. **b** Real-time PCR showed that circRNA_09505 was decreased in sh-circRNA-transfected macrophages compared with controls. **c** Representative FISH picture showed that circRNA_09505 was primarily localized in the cytoplasm of macrophages. **d** Representative scanning picture of EdU showing increased proliferation of LV-circRNA_09505-transfected macrophages compared with control group. **e** CCK-8 showed that the proliferation of LV-circRNA_09505-transfected macrophages was increased. **f** Representative scanning picture of EdU showing decreased proliferation of sh-circRNA_09505-transfected macrophages compared with control group. **g** CCK-8 showed decreased proliferation of sh-circRNA_09505-transfected macrophages. **h** The cell cycle of LV-circRNA_09505-transfected macrophages was estimated by flow cytometry. **i** The cell cycle of sh-circRNA_09505-transfected macrophages was estimated by flow cytometry. We calculated cell percentages in G0/G1, S, and G2/M phases, respectively. All data represented three independent tests. *P < 0.05, **P < 0.01, ***P < 0.001.

The expression of circRNA_09505 in macrophages was significantly increased when cells were transfected by circRNA_09505-overexpressed lentivirus plasmids (LV-circRNA) in contrast to the LV–NC group (Fig. 2a). Reversely, the expression of circRNA_09505 in macrophages transfected by sh-circRNA plasmids was reduced compared with sh-NC group (Fig. 2b). As demonstrated by fluorescence in situ hybridization (FISH), circRNA_09505 was mainly localized in the cytoplasm of macrophages (Fig. 2c). As a result, circRNA_09505 might influence macrophage proliferation and functions by interacting with other polypeptides, proteins, or nucleic acids that possess biological activity in the cytoplasm. First, we investigated the effect of circRNA_09505 on macrophage growth in vitro. As evidenced by EdU and CCK-8, the proliferation of macrophages was significantly increased when circRNA_09505 was overexpressed, but decreased when circRNA_09505 was knocked down in macrophages (Fig. 2d–g). In addition, FACS showed that circRNA_09505 could also enhance the progression of the cell cycle of macrophages (Fig. 2h, i). Taken together, circRNA_09505 was capable of enhancing macrophage proliferation and cell-cycle progression in vitro.

### CircRNA_09505 regulated macrophage inflammatory response through circRNA_09505–miR-6089 ceRNA network

**Fig. 3:**
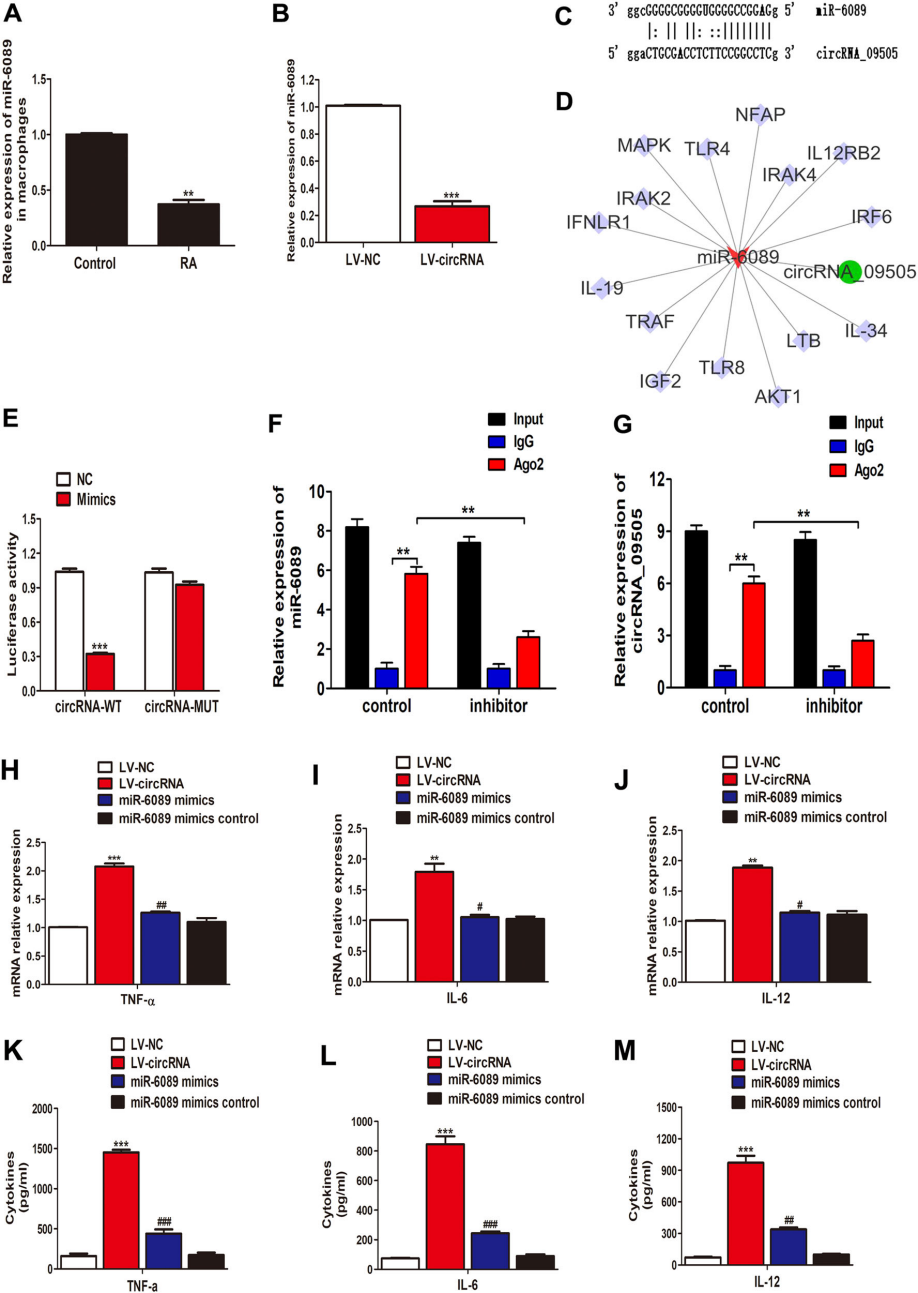
CircRNA_09505–miR-6089–mRNA network in macrophages. **a** Decreased miR-6089 expression in macrophages from RA patients compared with healthy controls (RA/Control: 21/18, **P < 0.01). **b** Reduced expression of miR-6089 in LV-circRNA_09505-transfected macrophages as evidenced by real-time PCR (***P < 0.001, data of three independent experiments). **c** Complementary bases of circRNA_09505 and miR-6089. **d** Targeted mRNAs of circRNA_09505–miR-6089 axis scanned in databases of Miranda and TargetScan. **e** Luciferase reporter assay demonstrated that miR-6089 was a targeted miRNA of circRNA_09505 (WT wild type, MUT mutant type, compared with normal control (NC) group, ***P < 0.001; representative data of three repeated tests in vitro). **f** RIP assay was performed using input, IgG, or anti-Ago2 antibodies. Relative expression of miR-6089 assayed by real-time PCR. **g** RIP assay and relative expression of circRNA_09505 determined by real-time PCR. Data of three independent assays. **P < 0.01. **h** Real-time PCR showed TNF-α mRNA expression in macrophages of four groups (cells treated by LV–NC plasmids, LV-circRNA plasmids, miR-6089 mimics plus LV-circRNA plasmids, and miR-6089 mimic controls plus LV–NC plasmids, respectively). **i** Real-time PCR showed IL-6 mRNA expression in macrophages. **j** Real-time PCR showed IL-12 mRNA expression in macrophages. **k** ELISA demonstrated TNF-α production in the supernatant of macrophages. **l** ELISA demonstrated IL-6 production in the supernatant of macrophages. **m** ELISA demonstrated IL-12 production in the supernatant of macrophages. **k**–**m** Compared with the LV–NC group, ***P < 0.001; compared with LV-circRNA group, ^#^*P* < 0.05, ^##^*P* < 0.01, ^###^*P* < 0.001; data of three independent tests.

Previously, we have demonstrated that miR-6089 was downregulated in PBMCs of RA patients and could regulate macrophage inflammatory response via TLR4^15^. Here, the expression of miR-6089 was also found in microphage in RA patients (Fig. 3a). The negative association between circRNA_09505 and miR-6089 was observed in circRNA_09505-overexpressed macrophages (Fig. 3b). In addition, miR-6089 was predicted to be a targeted miRNA of circRNA_09505 by biological informatics analysis. MiR-6089 was capable of specifically recognizing 13 bases of circRNA_0950 sequence (Fig. 3c). Figure 3d shows the potential circRNA_09505–miR-6089–mRNA network, which was scanned in databases of Miranda and TargetScan. Given these findings, we hypothesized that circRNA_09505 could regulate macrophages by sponging miR-6089 through ceRNA mechanism. Moreover, the luciferase reporter assay had supported this hypothesis (Fig. 3e). In addition, RIP assay showed that both circRNA_09505 and miR-6089 were elevated in the immunoprecipitates of the anti-Ago2 group (Fig. 3f, g). Both circRNA_09505 and miR-6089 inhibitors were reduced in miR-6089 inhibitor-treated group compared with the IgG group (Fig. 3f, g). Accordingly, circRNA_09505 could function as a miR-6089 sponge in macrophages. In the following experiments, we investigated the influence of circRNA_09505/miR-6089 on macrophage-mediated inflammation in RA pathogenesis. CircRNA_09505 promoted the expression of TNF-α, IL-6, and IL-12 mRNAs in macrophages (Fig. 3h–j). The protein level of these cytokines in the cultural supernatant of macrophages was also increased when circRNA_09505 was overexpressed in cells (Fig. 3k–m). However, the rescue tests using miRNA mimics showed that miR-6089 mimics could suppress the effect of circRNA_09505 on macrophage inflammation (Fig. 3k–m). Taken together, circRNA_09505 regulated macrophage inflammatory response via miR-6089 sponge in vitro through ceRNA mechanism.

### CircRNA_09505–miR-6089 ceRNA target-regulated AKT1 depending on IκBα/NF-κB signaling pathway in macrophages

**Fig. 4:**
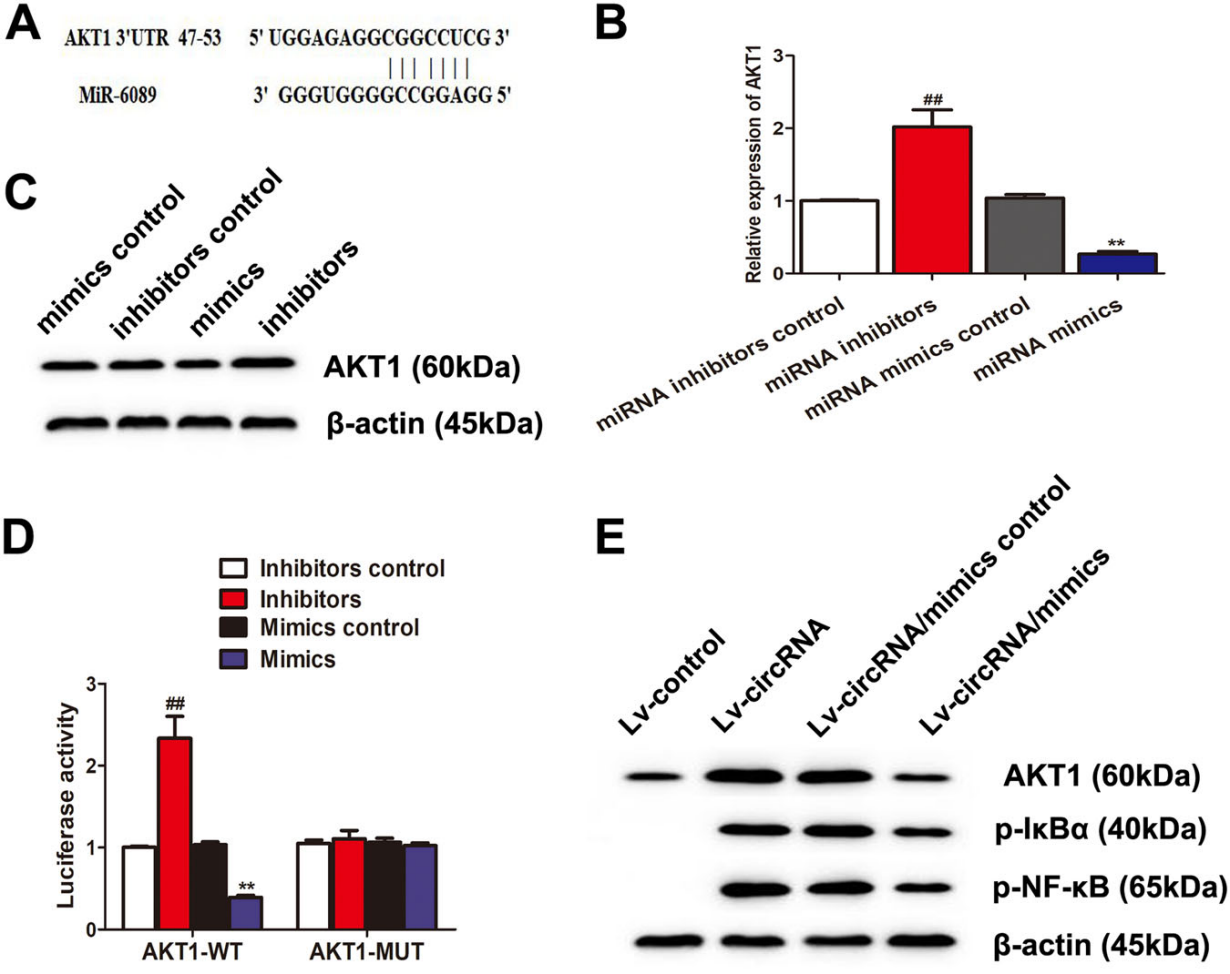
CircRNA_09505 regulated AKT1 via sponging miR-6089. **a** Complementary sequences of the 3′UTR of AKT1 recognized by miR-6089. **b** MiR-6089 inhibited the expression of AKT1 mRNA in macrophages as evidenced by real-time PCR. **c** Representative western blot estimating AKT1 protein. **d** Luciferase reporter assay demonstrated that AKT1 was a target of miR-6089, but the rescue test by use of miR-6089 inhibitors reversed its effect on AKT1. **e** The western blot analysis showed that circRNA_09505 increased the expression of AKT1, p-IκBα, and p-NF-κB in macrophages, while miR-6089 mimics partially restrained its effect through ceRNA mechanism. In contrast to miRNA mimic control group, **P < 0.01; compared with miRNA inhibitor control group, ^##^*P* < 0.01. Experiments were repeated at least three times.

To further investigate the targets of circRNA_09505–miR-6089 ceRNA network, we performed bioinformatics analysis in TargetScan database. Figure 4a shows that AKT1 was a potential targeted mRNA for miR-6089 because it had complementary bases in the 3′-untranslated region (UTR) recognized by miR-6089. Negative association between AKT1 and miR-6089 was found in macrophages (Fig. 4b, c). Besides, the luciferase reporter assay supported that miR-6089 could downregulate the expression of AKT1, whereas miR-6089 inhibitors could restrain its effect (Fig. 4d). Taken together, AKT1 was the targeted gene of miR-6089. Moreover, circRNA_09505 could promote the expression of AKT1, p-IκBα, and p-NF-κB in macrophages (Fig. 4e). However, miR-6089 mimics could partially rescue this effect via circRNA_09505/miR-6089 sponge (Fig. 4e). These findings demonstrated that circRNA_09505 could act as a ceRNA by acting as a miR-6089 sponge and regulate inflammatory response through AKT1/NF-κB signaling pathway in macrophages.

### Blocking circRNA_09505 in macrophages alleviated inflammation and joint damage in CIA mice

**Fig. 5:**
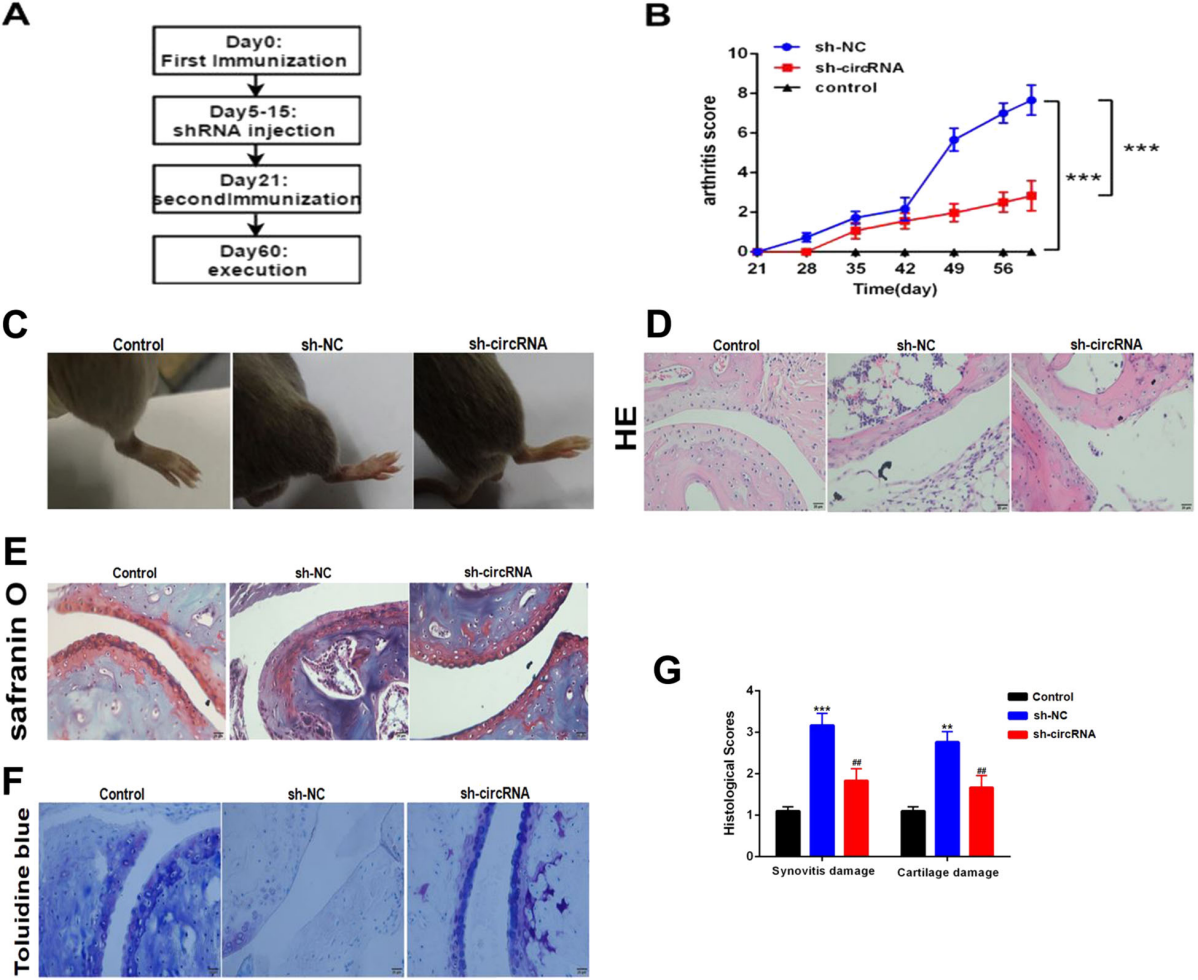
Knockdown of circRNA_09505 in macrophages prevented against arthritis in CIA mice. **a** The flowchart for constructing CIA mice model (eight mice for each group). **b** Blocking circRNA_09505 in macrophages reduced the mean arthritis score and prolonged the time of arthritis that first appeared in contrast to sh-NC-macrophage-treated CIA mice. **c** Representative pictures of CIA mice model. Less severe joint redness and swelling in sh-circRNA-treated CIA mice compared with sh-NC group. **d** Representative HE staining of knee joint sections showed decreased subchondral bone erosions, damages, and lymphocyte infiltration in sh-circRNA-transfected macrophage-treated CIA mice. **e** Representative Safranin O staining presented less knee joint injury and bone destruction in sh-circRNA-macrophage-administered CIA mice. **f** Representative Toluidine blue staining of knee joint sections showed that knockdown of circRNA_09505 protected the CIA mice from severe bone destruction. **g** The quantitative analysis showed less severe synovitis and cartilage damage of CIA mice when circRNA_09505 was blocked in macrophages. Compared with controls, **P < 0.01, ***P < 0.001; in contrast to sh-NC group, ^##^*P* < 0.01. At least three repeated experiments were conducted.

**Fig. 6:**
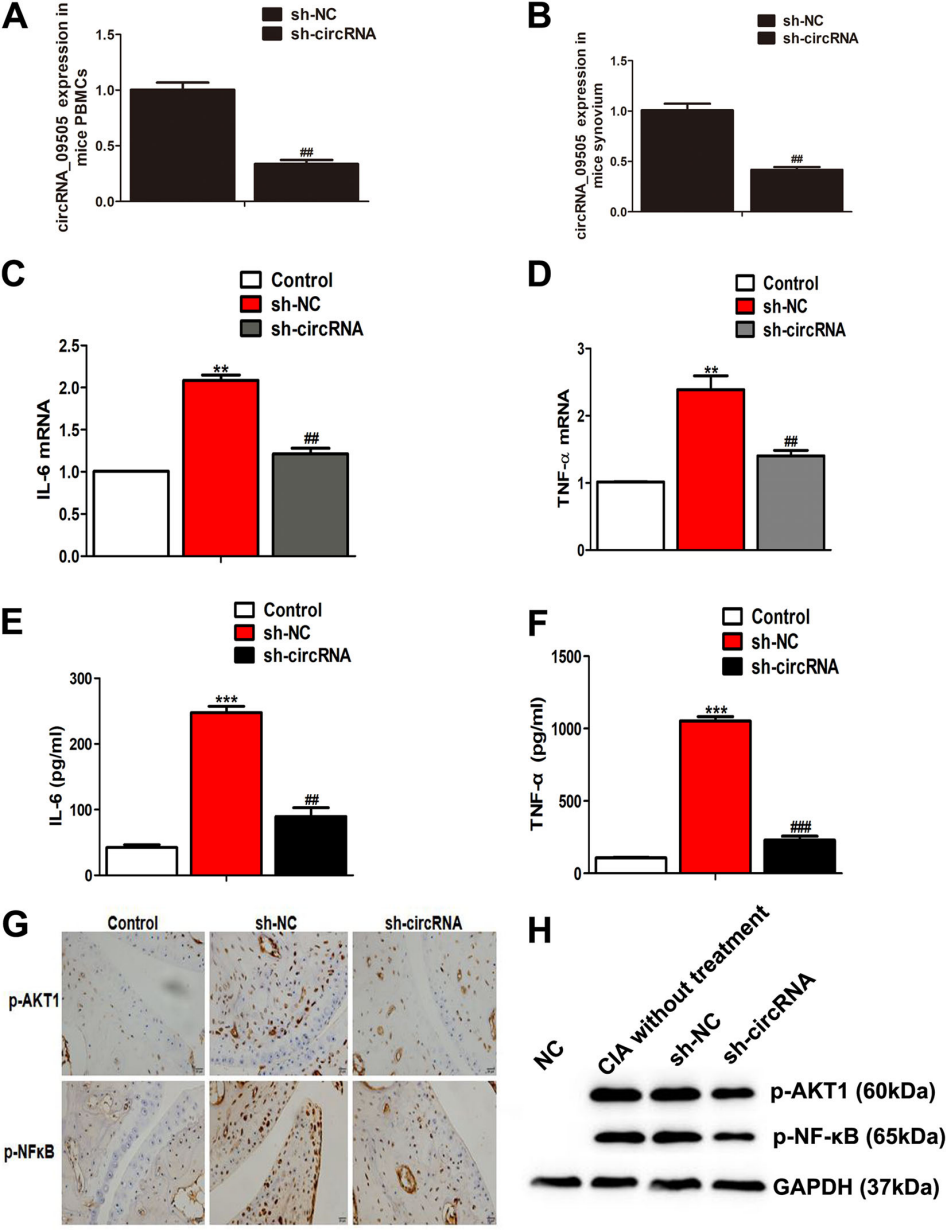
Knockdown of circRNA_09505 in macrophages alleviated inflammation via AKT1/NF-κB signaling pathway in CIA mice. **a** Decreased expression of circRNA_09505 in PBMCs of CIA mice treated with sh-circRNA plasmid- transfected Raw264.7 macrophages compared with CIA mice treated with sh-NC plasmid-transfected macrophages. **b** Reduced expression of circRNA_09505 in synovium of CIA mice determined by real-time PCR. **c** Knockdown of circRNA_09505 in macrophage-inhibited IL-6 mRNA expression in PBMCs from CIA mice. **d** Knockdown of circRNA_09505 in macrophage-inhibited TNF-α mRNA expression in PBMCs from CIA mice. **e** Knockdown of circRNA_09505 in macrophages decreased IL-6 in mouse serum. **f** Knockdown of circRNA_09505 in macrophages decreased TNF-α protein in mouse serum. **g** Representative immunohistochemistry sections showing decreased expression of p-AKT1 and p-NF-κB in mouse knee joint synovial tissues. **h** Representative western blot pictures showing reduced expression of p-AKT1 and p-NF-κB in mouse knee joint synovial tissue macrophages of CIA mice. Compared with normal controls (NC), **P < 0.01, ***P < 0.001; in contrast to sh-NC group, ^##^*P* < 0.01; ^###^*P* < 0.001. At least three repeated experiments were conducted.

CIA mice model was constructed following the flowchart in Fig. 5a. Blocking circRNA_09505 in macrophages significantly reduced the mean arthritis score and prolonged the time of arthritis that first appeared compared with the control mice (Fig. 5b). Less severe joint injury, reduced subchondral bone erosions, and lymphocyte infiltration in tissues were observed in sh-circRNA-transfected macrophage-treated mice (Fig. 5c, d). Knockdown of circRNA_09505 in macrophages protected CIA mice from severe bone destruction as seen in histomorphometric assays (Fig. 5e, f). Synovitis and cartilage damage of CIA mice were obviously alleviated when they were treated with circRNA_09505-blocked macrophages (Fig. 5g). circRNA_09505 was decreased in PBMCs and synovium of CIA mice administered with sh-circRNA plasmid-transfected macrophages (Fig. 6a, b). F4/80^+^ macrophages were less infiltrated in mice synovial tissues when they were treated with circRNA_09505 knocked-out macrophages (Supplementary Fig. 2). The expression of IL-6 and TNF-α mRNA in mice PBMCs was significantly decreased after blocking circRNA_09505 in macrophages (Fig. 6c, d). Cytokines of IL-6 and TNF-α in mouse serum were also obviously reduced in mice that were treated with circRNA_09505 knocked-down macrophages (Fig. 6e, f). TNF-α was decreased in mouse synovial tissue macrophages that were treated with circRNA_09505 knocked-down macrophages, while IL-4 in macrophages was increased (Supplementary Fig. 3). In addition, the expression of p-AKT1 and p-NF-κB in mouse synovial tissues was determined by immunohistochemistry. The phosphorylation and activation of AKT1 and NF-κB in joint synovial tissue macrophages were significantly inhibited in CIA mice treated with circRNA_09505-blocking macrophages (Fig. 6g, h). As a result, it could be concluded that circRNA_09505 could influence the inflammation of CIA mice in vivo. Blocking circRNA_09505 in macrophages could alleviate inflammation and joint damage in CIA mice.

## Discussion

A growing body of data have implicated that ncRNAs are key regulators for coding genes at transcriptional or post-transcriptional levels in a variety of autoimmune diseases including RA^7,12,16^. CircRNAs have been initially identified as ncRNAs and gradually highlighted in multiple physiological and pathophysiological processes^12^. A large body of data has suggested the pivotal role of circRNAs in regulating autoimmunity and inflammation. There is sufficient evidence that a number of circRNAs are aberrantly expressed in RA, which may participate in RA pathogenesis^17,18^. Our study shows the profile of circRNAs in PBMC samples from RA patients by RNA sequencing. Besides, we have first demonstrated that circRNA_09505 is the most significantly increased circRNA in RA PBMCs, which can function as miR-6089 sponge and regulate macrophage inflammation by targeted regulation of AKT1 in vitro. Moreover, blocking circRNA_09505 in macrophages alleviates inflammation and joint damage in CIA mice in vivo. As a result, circRNA_09505–miR-6089 ceRNA network would serve as novel targets for RA diagnosis and treatment.

CeRNA is well known as a mechanism about the interaction between different RNAs. It has been well established that miRNA can result in gene silencing by binding to mRNA, whereas ceRNA can regulate mRNA and affect gene silencing caused by miRNA by competing with it^19^. CeRNA cross talk has been documented to contribute to disease pathogenesis, such as cancer, inflammation, and autoimmunity^20,21^. Mounting studies have shown the evidence that a variety of RNAs, including circRNAs, lncRNAs, mRNAs, and pseudogenes, can function as miRNA sponge via ceRNA mechanism in various biologic processes. In particular, some available studies have reported the sequencing and microarray technologies to investigate the dysregulated transcripts in certain cells or whole blood of RA patients, some of which are demonstrated to be functional ceRNAs in RA^22,23^. We have previously found that lncRNA HIX003209 is dysregulated in RA, which functions as ceRNA for miR-6089 and regulates inflammation by targeting TLR4 in macrophages^24^. Accordingly, ncRNAs play a crucial role in RA pathogenesis by ceRNA cross talk.

CircRNAs belong to a family of endogenous ncRNAs without 5′ caps or 3′ tails, which have been implicated in inflammation and immune regulation. Apart from interacting with molecules with biological activity directly, a number of circRNAs have also been confirmed to function as ceRNA in cancer, inflammation-related, or autoimmune diseases^25–27^. Although several published studies have identified the specific profile of circRNAs in peripheral blood cells of RA patients, whether those circRNAs can function as ceRNA for the dysregulated miRNAs in RA is not clear yet. We, for the first time, demonstrate that dysfunction of circRNA_09505 contributes to RA by regulating macrophage proliferation, cell cycle, and inflammatory response. In addition, it is involved in macrophage inflammation through circRNA_09505–miR-6089 ceRNA network. CircRNA_09505 can promote AKT1 expression and NF-κB activation in macrophages in vitro, although miR-6089 mimics can partially rescue this effect via circRNA_09505/miR-6089 sponge. Moreover, knockdown of circRNA_09505 in macrophages alleviates inflammation and joint damage in CIA mice in vivo. As a result, circRNA_09505 may be a potential target for RA treatment in combination with macrophage therapy. Nonetheless, more investigations are warranted to apply it to RA treatment in the future.

Macrophages are key cells that participate in inflammatory and immune reactions, which play a vital role in RA. Elevated level of macrophages can infiltrate in the inflamed synovium tissue and cause inflammatory lesions and joint damage in RA^3^. Some circRNAs are reported to be specifically expressed in macrophages and affect their differentiation, infiltration, activation, and function in particular tissues^28–30^. There are different expression patterns of circRNAs in diverse polarized macrophages primarily including pro-inflammatory M1 and anti-inflammatory M2 macrophages^28^. However, the mechanism by which circRNAs regulate macrophage polarization and function particularly in RA remains obscure. The present study shows the specific expression profile of circRNAs in PBMCs from RA patients, while the expression of circRNAs in polarized macrophages and their molecular mechanism is not elucidated. Investigating the mechanism that drives macrophage polarization toward M1 or M2 phenotypes is worthy to be done, because depletion of macrophages with pro-inflammatory phenotype like circRNA_09505 from the inflamed tissue is a promising therapeutic strategy for RA. In this study, we have demonstrated the modifying effect of circRNA_09505 on macrophages in vitro. CircRNA_09505 aggravates macrophage inflammation by promoting the generation of inflammatory cytokines TNF-α, IL-6, IL-8, IL-12, and IL-1β, which are most commonly dysregulated cytokines in RA. In addition, TNF-α in synovial macrophages is significantly decreased when CIA mice are treated with circRNA_09505-blocking macrophages through tail-vein injection. However, whether circRNA_09505 regulates macrophage differentiation and polarization in vivo is unclear. Accordingly, more studies, particularly in vivo research, are encouraged to elucidate the role of circRNA_09505 in macrophage differentiation and function in the future.

To summarize, circRNA_09505 regulated macrophage inflammation by functioning as a ceRNA for miR-6089 through AKT1/NF-κB signaling pathway. Blocking circRNA_09505 in macrophages can reduce inflammation and joint damage in CIA mice. These findings show strong evidence supporting the use of circRNA_09505 as a target for RA immune therapy. Nevertheless, more mechanisms and function-associated experiments are warranted to explore it as a useful treatment strategy for RA in the future.

## Materials and methods

### Patients, controls, and animals

There are 36 initially diagnosed and untreated RA patients and 30 age- and gender-adjusted healthy controls from the First Affiliated Hospital of Weifang Medical University. The characteristics of the participants are shown in Table 2. They have read and signed the written informed consent before peripheral blood sample collection. The present study is approved and supervised by the Institutional Ethics Committee of the First Affiliated Hospital, Weifang Medical University. We extract PBMCs from patients and controls according to the protocol of human lymphocytes isolation solution (TBD, Tianjin, China) by gradient centrifugation at 1800*g* for 30 min. PBMCs and serum samples are also stored at −20 °C for subsequent use. In addition, peripheral blood monocytes are isolated from 21 RA patients and 18 age- and gender-adjusted healthy controls by CD14 microbeads (Miltenyi Biotec, San Diego, USA) according to the protocol. C57BL/6 (8 weeks old) male mice are purchased from Pengyue Experimental Animal Breeding Co., Ltd. (Jinan, Shandong Province, China), which are used to construct CIA mice model. Mice were randomly divided in this study. All animal experiments are carried out based on the Guideline of Institutional Animal Care and Use Committee. About 4 mg/ml bovine type II collagen (Chondrex, Washington, USA) is dissolved in 50 mM acetic acid and emulsified with Freund’s complete adjuvant (Sigma-Aldrich, USA) of equal volume by repeated suction. Eight mice in each group are immunized by use of 100 µl (4 mg/ml) of emulsion solution through intradermal injection at the base of the tail (day 0). Booster injection using 100 µl of emulsion solution is conducted by intradermal injection at another site of the tail base on day 21. In the treatment study, mice with the score reaching six are randomly separated into sh-circRNA-treated Raw264.7 macrophage group and sh-NC-treated macrophage group. Mice were intravenously injected with plasmid-transfected macrophages through the tail vein on days 5 and 15, respectively. The longest time for mice arthritis estimation is 60 days from the first immunization. Mouse PBMCs are isolated from eyeball blood using mouse lymphocytes isolation solution (TBD, Tianjin, China). Synovial macrophages are isolated from mouse joints according to the protocol of mouse tissue lymphocytes isolation solution (TBD, Tianjin, China) and anti-mouse F4/80 monoclonal antibody (Miltenyi Biotec, San Diego, USA). All samples and isolated cells are stored at −20 °C for determinations. Animal studies are approved by the Animal Ethics Committee of Weifang Medical University.

**Table 2:** Summary characteristics of RA patients and controls.

	Control (30)	RA (36)
Age (yrs)	23–60	25–63
Sex (M/F)	5/20	6/22
Tobacco smoking (yr)	15.0 ± 2.7	18.1 ± 3.2
Alcohol consumption (yr)	9.2 ± 1.5	10.3 ± 2.0
ESR (mm/h)	12.0 ± 1.8	48.1 ± 2.2
CRP (mg/L)	7.0 ± 1.6	40.9 ± 3.2
RF (IU/ml)	10.9 ± 2.0	71.5 ± 7.6

### RNA sequence

PBMC samples from three RA and three healthy controls were analyzed by high-throughput sequencing by Oebiotech Company (Shanghai, China) to screen the aberrantly and specially expressed circRNAs in RA. The co-expression of mRNAs in PBMCs from RA patients was also estimated. Prediction of potential target genes and signaling pathway of circRNAs were also adjusted by bioinformatics analysis in databases of Miranda and TargetScan.

### Cell culture and transfection

PMA (100 nM) was used to stimulate THP-1 for 48 h to make them differentiate into macrophage-like cells. THP-1 and Raw264.7 macrophages were cultured in DMEM (Sigma-Aldrich, USA) with 10% fetal bovine serum (Gibco, USA) at 37 °C, 5% CO_2._ MiRNA inhibitors, mimics, and controls are purchased from Ruibo Biosciences (Guangzhou, China). PcDNA3.1 lentivirus products LV-circRNA, sh-circRNA, and the corresponding negative controls are constructed in 293T cells using Lipofectamine 2000 (Invitrogen, NY, USA). Polybrene reagent is used to transfect macrophages. Stably transfected cells are finally verified by quantitative real-time PCR.

### Quantitative real-time PCR

The expression profile of circRNA in PBMCs of RA patients is screened by RNA sequencing in Oebiotech Company (Shanghai, China). Quantitative real-time PCR is applied to confirm the differentially expressed circRNAs and miR-6089 in RA. TRIzol (Invitrogen, CA, USA) is used for RNA isolation. About 0.5 µg of RNAs are used as a model for cDNA synthesis according to the protocol of Takara RT kit (Tianjin, China). About 5 ng of cDNAs are used as templates for PCR by use of Takara SYBR Green Mastermix kit (Tianjin, China). TaqMan miRNA real-time PCR kit (ThermoFisher Scientific, USA) is applied to determine miR-6089 expression by the protocol. Primers for human genes are listed as follows: CircRNA_09505, (F): GTGTGACCTCCACAGCTACC, (R): TCACCAGACACACTGTGAGG; IL-6, (F): 5′–3′ TGAACTCCTTCTCCACAAGC, (R): 5′–3′ CTGAAGAGGTGAGTGGCTGT; TNF-α: (F): 5′–3′ ATGTGGCAAGAGATGGGGAA, (R): 5′–3′ CTCACACCCCACATCTGTCT; IL-12, (F): CTTGGTTTTCCCTGGTTTTT, (R): CTTTGACTTGGATGGTCAGG; AKT1, (F): GACGGGCACATTAAGATCAC, (R): TGAGGATGAGCTCAAAAAGC; GAPDH, (F): AAGGAAATGAATGGGCAGCC, (R): TAGGAAAAGCATCACCCGGA.

### Cell proliferation and cell-cycle assay

In brief, 2 × 10^5^/well THP-1 macrophages are seeded into cell culture plates for 12, 24, and 48 h. Cell counting kit-8 (CCK-8) kit (Vazyme Biotech, Nanjing, China) and 5-ethynyl-2′-deoxyuridine (EdU) kit (RiboBio, Guangzhou, China) are used to determine the proliferation of LV-circRNA, sh-circRNA, and control macrophages. After RNase treatment, the cell cycle is estimated by FACSCalibur (BD Biosciences, San Jose, CA, USA) incubating with propidium iodide (Keygen Biotech) at 37 °C for 30 min. All experiments are performed at least three times.

### Cytokine assay

Human IL-6, TNF-α, IL-12, IL-1β, and IL-8 in the supernatant of macrophages are measured by Enzyme-linked Immunosorbent Assay (ELISA) according to the protocol of kits (R&D Systems, USA). IL-6 and TNF-α in mouse serum samples are determined by ELISA kits based on the manufacturer’s instructions. Anti-mouse IL-4 and TNF-α monoclonal antibodis (eBioscience, San Diego, California, USA) were used to detect the intracellular cytokines of mouse synovial macrophages by flow cytometry.

### Western blot

Cells are treated with cell lysis buffer containing 1ul of protease inhibitor and 1ul of phenylmethanesulfonyl fluoride. After denaturation, protein samples are subjected to SDS PAGE for isolation and then are transferred to PVDF membranes by electroblotting. Primary antibodies of AKT1, p-AKT1, p-IκBα, p-NF-κB, 𝛽-actin, and GAPDH (Cell Signaling Technology, MA, USA) are used to incubate with PVDF membranes, which are subsequently incubated with HRP-conjugated secondary antibodies (Proteintech, USA). The enhanced chemiluminescence kit (Cell Signaling Technology, MA, USA) is applied for detection.

### Hematoxylin–eosin (H–E), toluidine blue, and safranin O staining and immunohistochemistry (IHC)

Mice joint tissues are decalcificated by use of 10% EDTA, which is then embedded in paraffin for slides. H–E, Toluidine blue, and Safranin O (Solarbio) of knee joint sections are performed for detection of joint and cartilage damages. IHC was also carried out as previously described^31^. The slides are incubated overnight with p-AKT1 and p-NF-κB antibodies (Cell Signaling Technology, MA, USA).

### Fluorescence in situ hybridization (FISH) assay

The location of circRNA_09505 in macrophages is determined by FISH. Macrophages are fixed with 4% paraformaldehyde and gradiently dehydrated with ethanol. Fluorescent-labeled probe for circRNA_09505 is applied during hybridization. We use DAPI (Beyotime, Shanghai, China) to stain the nucleus of macrophages.

### Luciferase reporter assay

293T cells are used to estimate the targets of circRNA_09505 and miR-6089. Cells are transfected with miRNA mimics, inhibitors, AKT1-WT, AKT1-MUT, circRNA-WT, circRNA-MUT, or the corresponding plasmids with lipofectamine 2000. Macrophages are lysed and the luciferase activity is determined by Picagene Dual SeaPansy luminescence kit (Toyo Inc., Japan) based on the manufacturer’s instructions as reported previously.

### RNA-binding protein immunoprecipitation

RIP is preformed as described previously^24^. In brief, we perform experiments according to the protocol of Magna RIP Kit (Millipore, Bedford, USA). Cell lysates are immunoprecipitated using Ago2 antibody or IgG. The expression of circRNA_09505 and miR-6089 in immunoprecipitates is detected by real-time PCR as previously described, respectively.

### Statistical analysis

The arthritis scores are analyzed by Mann–Whitney *U* test between two groups. For other analyses, one-way ANOVA or Student’s t test is applied for estimation. SPSS 16.0 (SPSS Inc., Chicago, USA) and Graphpad 5.0 software are used. *P* < 0.05 is regarded to be statistically significant.

## Supplementary information

Supplementary Figure Legends

Supplementary Figure 1

Supplementary Figure 2

Supplementary Figure 3

## Data Availability

All data are available upon request of the corresponding author.
