# Supplementary material for: CircRNA_09505 aggravates inflammation and joint damage in collagen-induced arthritis mice via miR-6089/AKT1/NF-κB axis
Source: Cell Death Dis. 2020 Oct 7;11(10):833. doi: 10.1038/s41419-020-03038-z (PMC7542153; doi:10.1038/s41419-020-03038-z)
Supplement: Supplementary file 1 — Supplementary Figure Legends [file 41419_2020_3038_MOESM1_ESM.doc]

**Supplementary figure legends**

**Suppl. Figure 1 GO/KEGG pathway enrichment**

(A) GO: Biological Process; (B) GO: Molecular Function; (C) GO: Cellular Component; (D) KEGG Pathway.

**Suppl. Figure 2 CircRNA_09505 functioned as a ceRNA for miR-6089 and regulated IL-1β and IL-8 expression in macrophages**

(A) Real-time PCR results of IL-1β and IL-8 mRNA expression in macrophages. (B) ELISA: IL-1β and IL-8 in supernatant of macrophages. (compared with LV-NC group, ***, *P* < 0.001; compared with LV-circRNA group, ##, *P* < 0.01, ###, *P* < 0.001; Data of three repeated experiments)

**Suppl. Figure 3 Knockdown of circRNA_09505 in macrophages reduced macrophages infiltration and cytokines expression in synovial tissue macrophages of CIA mice**

(A) Flow cytometry:  F4/80^+^ macrophages infiltration in synovial tissue macrophages of CIA mice. (B) Flow cytometry:  TNF-α and IL-4 expression in synovial tissue macrophages of CIA mice.
