# Supplementary figures and images for: CircRNA_09505 aggravates inflammation and joint damage in collagen-induced arthritis mice via miR-6089/AKT1/NF-κB axis
Source: Cell Death Dis. 2020 Oct 7;11(10):833. doi: 10.1038/s41419-020-03038-z (PMC7542153; doi:10.1038/s41419-020-03038-z)

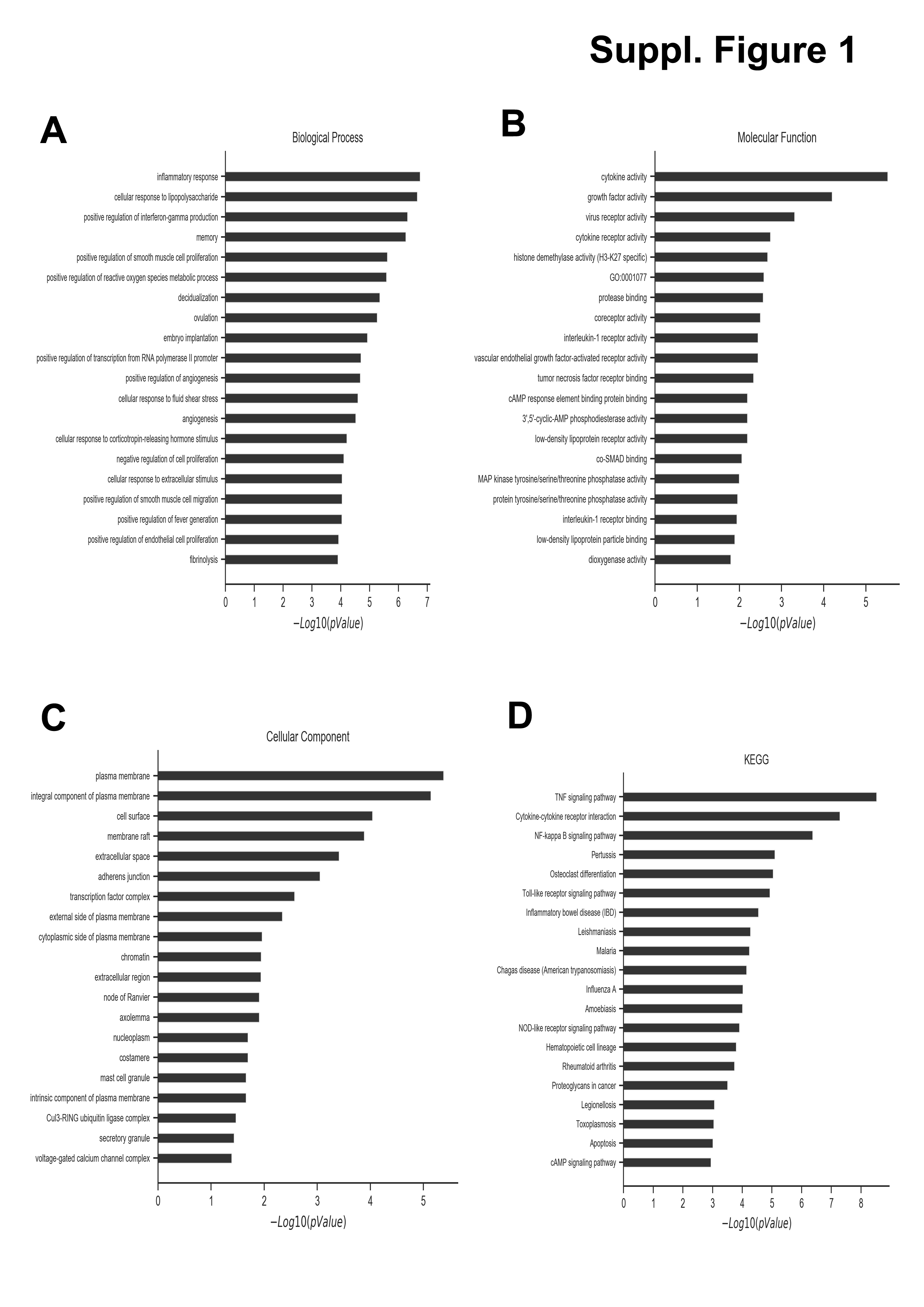

Supplement: Supplementary file 2 — Supplementary Figure 1 [file 41419_2020_3038_MOESM2_ESM.tif]

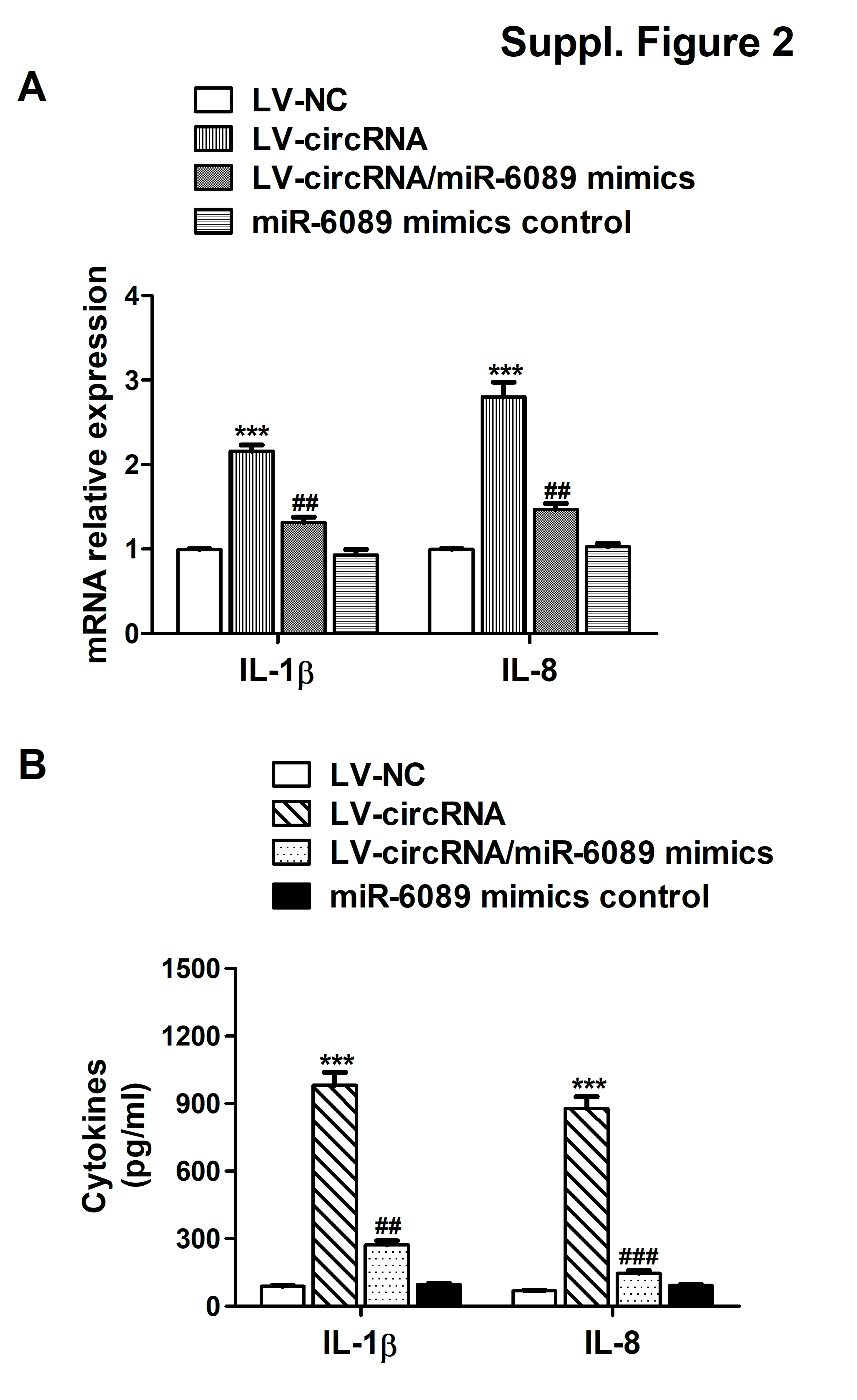

Supplement: Supplementary file 3 — Supplementary Figure 2 [file 41419_2020_3038_MOESM3_ESM.tif]

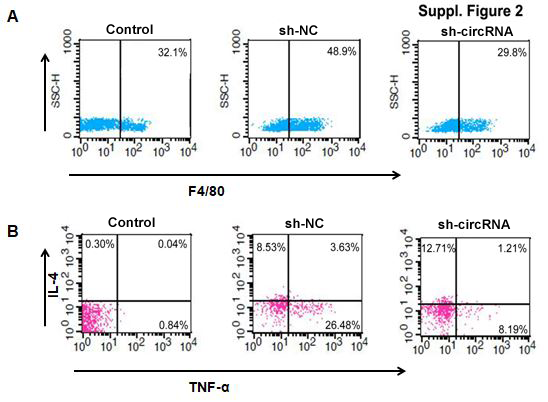

Supplement: Supplementary file 4 — Supplementary Figure 3 [file 41419_2020_3038_MOESM4_ESM.tif]
